# Septicemia due to *Streptococcus dysgalactiae* subspecies *dysgalactiae* in vampire bats (*Desmodus rotundus*)

**DOI:** 10.1038/s41598-018-28061-1

**Published:** 2018-06-27

**Authors:** Mateus de Souza Ribeiro Mioni, Fernando Favian Castro Castro, Luisa Zanolli Moreno, Camila Michelle Apolinário, Lais Dario Belaz, Marina Gea Peres, Bruna Letícia Davidé Ribeiro, Maria José da Silva Castro, Adriano Martison Ferreira, Adriana Cortez, Andrea Micke Moreno, Marcos Bryan Heinemann, Jane Megid

**Affiliations:** 1 Universidade Estadual Paulista “Júlio de Mesquita Filho”, Botucatu, São Paulo Brazil; 2 Universidad Antonio Nariño, Popayan, Cauca Colombia; 3 Universidade de São Paulo, São Paulo, Brazil; 4 Universidade de Santo Amaro, São Paulo, São Paulo Brazil

## Abstract

Beta-hemolytic *Streptococcus dysgalactiae* is a well-known pathogen for a wide range of animals and humans. Two subspecies are recognized: (i) *equisimilis*, associated to disease in horses and humans, and (ii) *dysgalactiae* mainly isolated from animal illness with only a few humans’ cases. This study describes the isolation and characterization of *Streptococcus dysgalactiae* subsp. *dysgalactiae* (SDSD) from vampire bats, maintained in captivity for research proposes. Animals presented neurologic, respiratory and gastroenteric symptoms and sudden death. Beta-hemolytic Gram-positive cocci were isolated in blood agar plates and further characterized as Lancefield group C. All isolates were identified as *S*. *dysgalactiae* by matrix-assisted laser desorption/ionization-time of flight (MALDI-TOF) mass spectrometry and subspecies *dysgalactiae* was confirmed by 16S rRNA sequencing and phylogenetic analysis. Genotyping through SE-ALFP resulted in three profiles (A1–A3) with one bat being infected by profiles A1 and A3. This is the first report of SDSD causing illness in bats and especially in *Desmodus rotundus* species.

## Introduction

The *Streptococcus* genus comprises Gram-positive, catalase-negative, cytochrome-negative, aerotolerant anaerobe and nonmotile bacteria^1,2^. The genus is divided into seven groups with *Streptococcus dysgalactiae* belonging to the pyogenic group^2^. Most of the pyogenic streptococci are considered pathogenic for humans and animals and are characterized by β-hemolysis due to the activity of hemolysins, streptolysin O and especially streptolysin S. They can also be characterized by polysaccharide variation detectable by the Lancefield method^2^.

The *Streptococcus dysgalactiae* is further divided into the subspecies *S*. *dysgalactiae* subsp. *dysgalactiae* (SDSD) and *S*. *dysgalactiae* subsp. *equisimilis* (SDSE)^3,4^. SDSE includes isolates from human and animals with strong β-haemolysis and is inserted into Lancefield serogroups A, C, G, and L, while SDSD is isolated mainly from animals such as cattle, dogs, pigs and other species^3,5–9^, with only a couple of human cases associated with articular infection after a total knee arthroplasty^10^, and cellulitis associated with the preparation of raw seafood^11^, present α-, β-, or nonhemolytic activity, and belong to Lancefield groups C and L^2–4^.

The *Streptococcus* genus has been isolated from different bats species^12–16^. The oral cavity of bats, as other mammals, is colonized by a wide range of streptococcal species mostly belonging to the mutans group^12^. In rectal swab of four different flying fox species (*Pteropus* sp.), Heard *et al*.^14^ detected α-hemolytic and group D *Streptococcus*. Helmick *et al*.^15^ also associated the presence of α-hemolytic *Streptococcus* to the death of two captive megachiropteran bats due to pneumonia. The *S*. *dysgalactiae* species is poorly studied in bats, even though it has already been isolated from the gut of healthy *Desmodus rotundus*^13^.

Here we present the isolation and characterization of *Streptococcus dysgalactiae* subsp. *dysgalactiae* from five vampire bats with clinical signs of encephalitis, pneumonia, and sudden death. This is the first report of SDSD causing septicemia and encephalitis in *Desmodus rotundus*.

## Results

### Clinical signs

**Figure 1:**
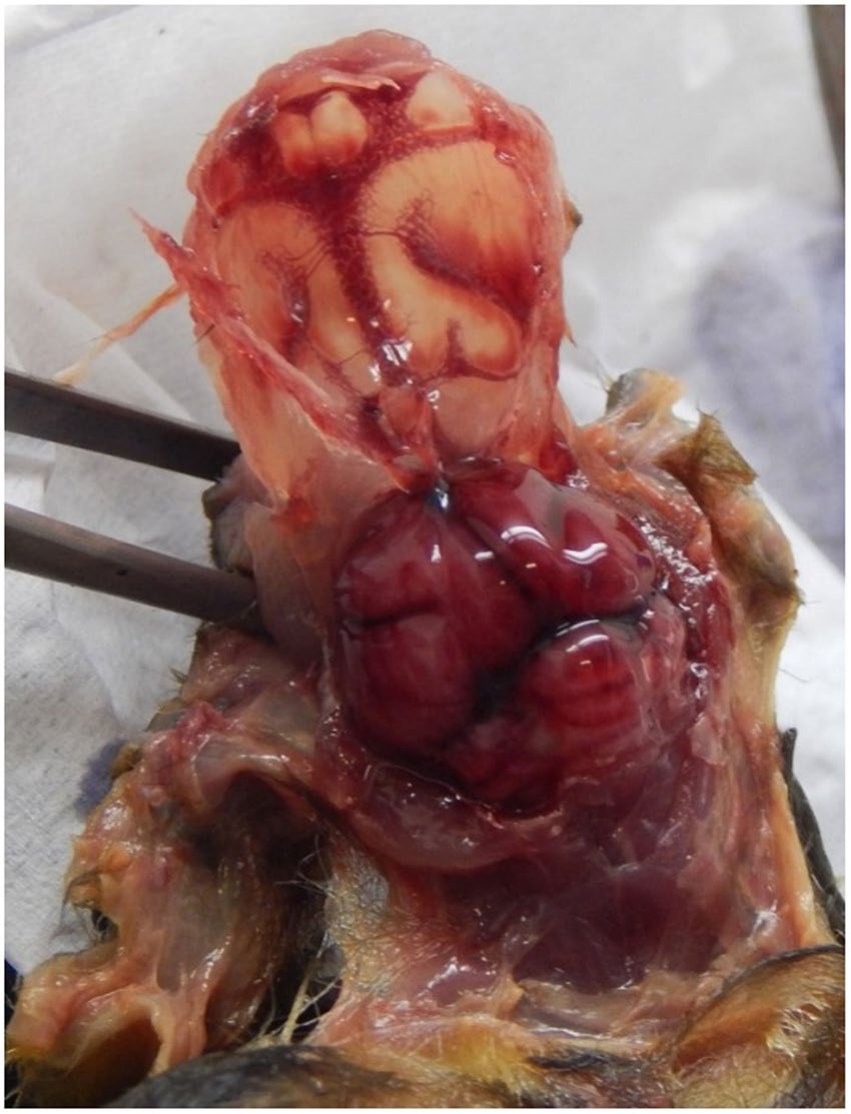
At necropsy, severe brain congestion of a vampire bat ill from *Streptococcus dysgalactiae* subsp. *dysgalactiae* infection.

From the 20 *Desmodus rotundus* that were kept in captive, 18 of them became ill and presented various signs, such as anorexia (1/18), neurologic symptoms (paralysis) (8/18), pneumonia (1/18), and sudden death (8/18), within 13 weeks, on average, of captivity and observation. At post-mortem necropsy, encephalitis and congestion of different organs were observed (Fig. 1). The brain congestion in accordance with the neurologic symptoms presented suggested the possibility of rabies, and also because some cases of rabies in cattle had been reported in the area of capture. All animals were negative for rabies in real-time PCR (data not shown). In attempt to elucidate the causative agent, bacterial isolation was performed from different tissues (lung, liver, intestine, brain) of five bats.

### Characteristics of the isolates

Gram-positive, catalase-negative, coccus-shaped organisms were isolated from all clinical samples of different organs suggesting bacteremia. Grey beta-hemolytic colonies were isolated on blood agar after 24 h of incubation at 37 °C. All isolates were characterized as Lancefield group C (Table 1).

**Table 1:** Streptococcal isolates from different individuals and organs of vampire bats, São Paulo state, Brazil.

Strains^*^	Species and subspecies	Clinical signs	Date of death	City of origin
4Li	*S*. *dysgalactiae* subsp. *dysgalactiae*	Sudden death	04/05/2016	Anhembi
4I				
4L				
5Li	*S*. *dysgalactiae* subsp. *dysgalactiae*	Sudden death	04/05/2016	Botucatu
5I				
5L				
13Li	*S*. *dysgalactiae* subsp. *dysgalactiae*	Neurologic symptoms	30/04/2016	Bofete
13I				
13L				
14I	*S*. *dysgalactiae* subsp. *dysgalactiae*	Neurologic symptoms	10/05/2016	Bofete
14L				
17L	*S*. *dysgalactiae* subsp. *dysgalactiae*	Sudden death	04/05/2016	Bofete

^*^The number indicates the animal and the letters indicate the organs of isolation. L - lungs; I - intestine; Li - liver.

### MALDI-TOF MS identification

The *Streptococcus dysgalactiae* species were identified by MALDI-TOF MS for all isolates. Considering this result, 16 S rRNA sequencing and phylogenetic analysis were proceeded for subspecies identification.

### 16 S rRNA sequencing and phylogenetic analysis

**Figure 2:**
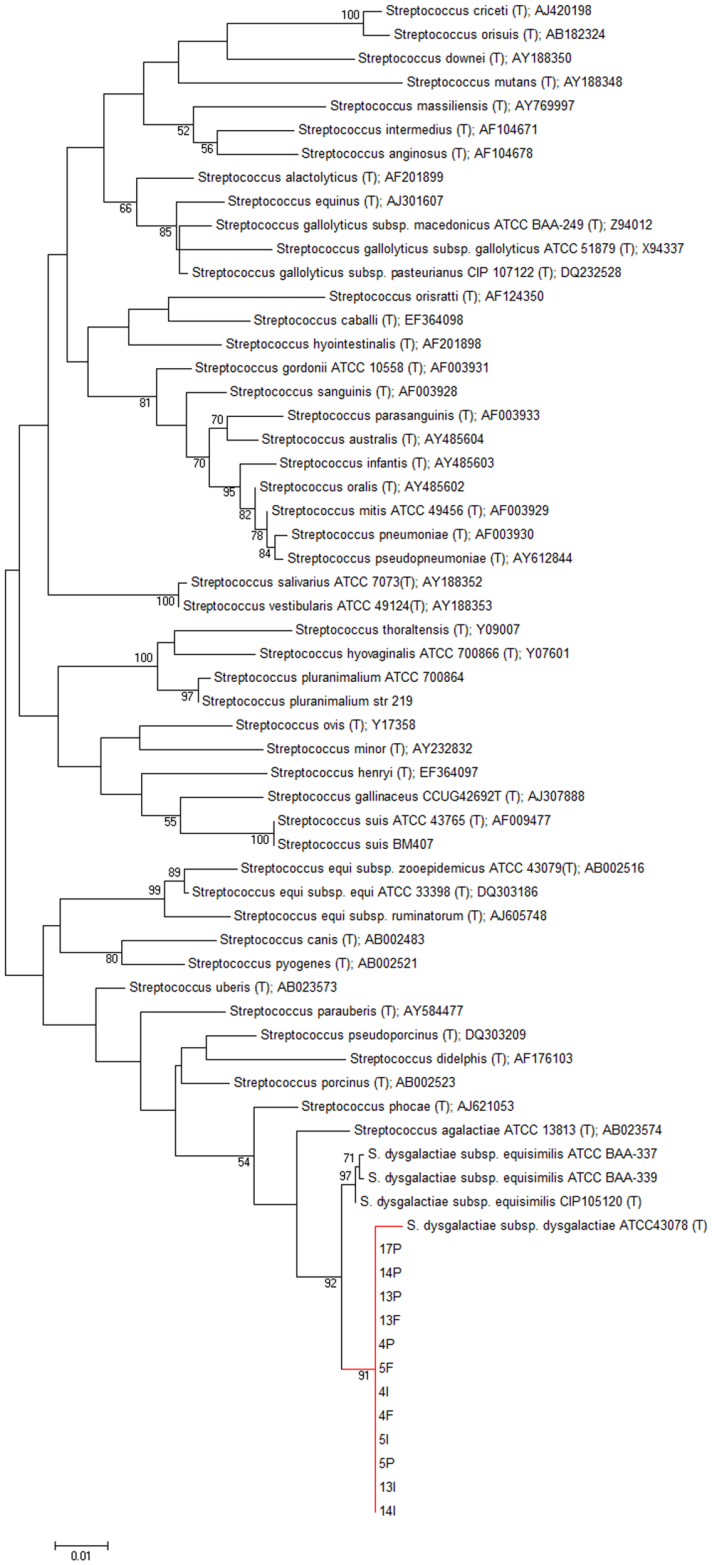
Phylogenetic tree based on the 16S rRNA nucleotide sequences for *Streptococcus dysgalactiae* isolated from vampire bats. The bootstrap values are presented at the corresponding branches.

The subspecies identification was obtained through 16 S rRNA sequencing and phylogenetic analysis. All isolates were identified as *S*. *dysgalactiae* subspecies *dysgalactiae* (>99% sequence identity with *S*. *dysgalactiae* subsp. *dysgalactiae* strain ATCC43078) (Fig. 2).

### SE-AFLP analysis

**Figure 3:**
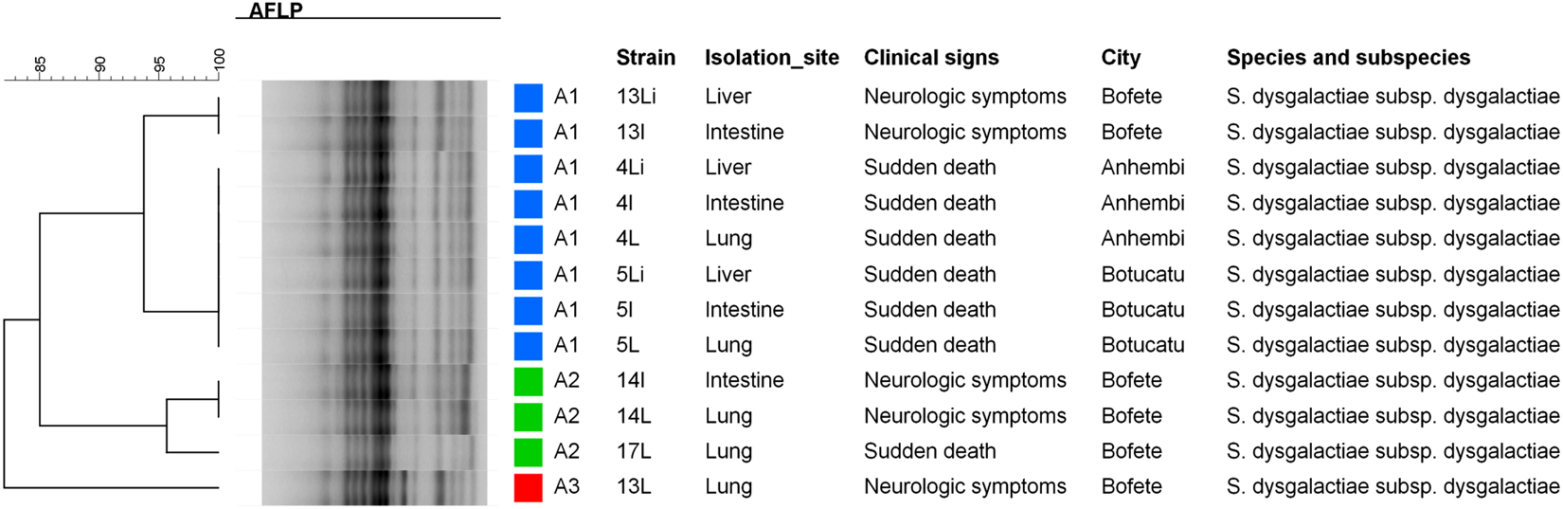
Dendrogram showing the relationship among the SE-AFLP patterns from *Streptococcus dysgalactiae* isolated from vampire bats.

SE-AFLP genotyping of the 12 studied isolates resulted in three profiles (A1–A3) with high genetic similarity (>80%) (Fig. 3). The gel with SE-AFLP profiles of SDSD can be found as Supplementary Fig. S1. The A1 cluster comprised eight isolates originated from three animals from different cities, while the A2 profile comprised three strains isolated from two animals of the same city. The 13L isolate presented a distinct fingerprint pattern from the other studied strains, even though it originated from the same animal of the 13Li and 13I isolates (A1 cluster).

## Discussion

Streptococci are predominant members of the commensal microbiota of the mucous membranes of the human oral cavity and to a lesser extent of the nasopharynx^2^. Although some streptococcal commensals have no significant record of disease transgressions, others streptococci have a lot of virulence factors, acting as pathogens capable of spread and of initiating infection in immunocompromised individuals^2,9,17^. The disease-associated streptococcal species may also occur in the asymptomatic host in which they are maintained in a sub-disease threshold load^2^. Changes in the pathogen population or to the host environment, such as decrease of competitors, acquisition of virulence factors, immunosuppression, and dietary changes, can affect the host-pathogen relationship and initiate the disease process^2,18^.

This could explain the observed outbreak, considering that the stress provoked by the environmental changes and animals diet could originate a momentary immunosuppression and favor the pathogen development. As the feeding blood was not contaminated, the *S*. *dysgalactiae* was probably introduced by a healthy bat harboring the pathogen, previously to the capture, that started to manifest the disease and excrete the bacteria due to the stressful changes. The horizontal transmission is sustained by the SE-AFLP genotyping that demonstrates high genetic similarity within the studied isolates and also the A1 cluster comprised isolates from three different cities that died in different days.

The symptoms described in the observed bats are common to streptococcal infection and especially to *S*. *dysgalactiae*. Also, in captive bats pneumonia has already been related to α-hemolytic *Streptococcus* infection^15^. As observed here, sudden death and organ congestion related to SDSD β-hemolytic Lancefield group C infection were also evidenced in puppies^6^. *Streptococcus dysgalactiae* is reported as a cause of bacteremia, meningoencephalitis, and mastitis in sheep^2^, and articular abscess in pigs^5^. *S*. *dysgalactiae* of group C occasionally causes lower limb cellulitis, meningitis, and bacteremia in humans^19–21^. β-hemolytic *S*. *dysgalactiae* of groups C and G can cause severe and recurring invasive infections^21^.

Among the mammalian class, bats are the most important reservoir of zoonotic pathogens and are estimated that they possibly may harbor several “missing zoonoses” that still were not detected^22^. For this reason, the order of Chiropteran is incriminated as of highest value for surveillance, especially in South and Central America and parts of Asia^22^. Furthermore, several pathogens that may affect humans such as *Bartonella* spp^23,24^., *Polyomaviridae*^25^, influenza A virus^26^, among several others^22^, and the isolation of pathogenic SDSD shows the probability of transmission of another pathogen by bats to animals and humans. The interface of bats and humans can occur in a variety of ways like incidental contact, degradation of the environment by humans, controlled contact for research proposes and predation^27^. The widespread distribution of vampire bats on the American continent, ranging from Mexico to southern South America^22,28,29^, encompasses around fourteen countries^29^ and shows that millions of people and animals are vulnerable to bats attacks in rural areas and also in places where the degradation of the environment alters the proximity between human and animals, and those scenarios are very common in the geographic areas where the vampire bat is found.

The *S*. *dysgalactiae* species identification was obtained through MALDI-TOF MS and subspecies *dysgalactiae* was only identified by 16 S rRNA sequencing and phylogenetic analysis. Even though MALDI-TOF MS has comparable discriminatory power to molecular techniques for bacterial species differentiation^30^ and presented high-confidence for β-hemolytic streptococci species identification^31^, this technique still does not enable proper *S*. *dysgalactiae* subspecies differentiation. For subspecies identification, the phenotypic characterization still causes confusion, as corroborated by our results since Lancefield group C β-hemolytic colonies could be allocated in both subspecies. Considering that the misclassification of β-hemolytic SDSD into subspecies *equisimilis* has been previously reported^4,6^ and was only solved by 16S rRNA analysis, the molecular techniques still are more appropriate for this differentiation level of *S*. *dysgalactiae*. Interestingly, Jensen & Kilian^4^ observed that all β-hemolytic SDSD originated from invasive infections, which also corroborates our results.

We present the first report of SDSD infection in vampire bats (*Desmodus rotundus*) causing septicemia and encephalitis. Even though the clinical relevance of SDSD for animal health further increases, its zoonotic potential remains unknown. Therefore, due to the importance of *S*. *dysgalactiae* subspecies, they should be properly identified by veterinary diagnostic laboratories. Also, the presence of SDSD in bats corroborates with the theory that bats harbor a lot of pathogens, which raises the question about the possible involvement of this species in the dissemination of this and others pathogens.

## Methods

### Animals

The study was designed for the study of serological evaluation of anti-rabies antibodies in bats as an epidemiological tool for the control of the disease in hematophagous chiropterans. Twenty Desmodus rotundus were kept in captive (authorization number 51231-1, issued by the Ministry of the Environment of Brazil) for research proposes. The bats were captured from natural caverns in three nearby cities from São Paulo state (Anhembi, Botucatu, and Bofete) (Table 1) and were considered as asymptomatic. The bats were housed and handled following the ethical principles adopted by Bioethics Commission of the Faculty of Veterinary Medicine and Animal Production of São Paulo State University (protocol number 85/2015) and all experimental protocols were approved by the same institution. Animals were fed with defibrinated blood that was collected in slaughterhouses, supplemented with cobalamin. The bats were observed for 137 days and within 85 days of the quarantine period, on average, 18 of them became ill presenting various signs (Table 1).

### Bacterial isolation and Lancefield group determination

Samples from different tissues (lung, liver, intestine, brain) of five bats were used in an attempt for bacterial isolation. The samples were inoculated on blood agar plates (CM0055, Oxoid, Hampshire, England) supplemented with 5% defibrinated sheep blood and incubated aerobically for 24 h at 37 °C. For isolated colonies, the Lancefield group was determined through commercial latex agglutination kit (Avipath Strep®, Omega Diagnostics, Scotland, United Kingdom) following manufacturer’s instructions. Samples of the blood used in the bats feeding were also inoculated on blood agar plates; however, no isolation was obtained.

### MALDI-TOF mass spectrometry identification

The obtained isolates were initially identified as *Streptococcus dysgalactiae* by matrix-assisted laser desorption/ionization-time of flight (MALDI-TOF) mass spectrometry. MALDI-TOF MS sample preparation, data processing, and analysis were done as previously described by Hijazin *et al*.^30^. Mass spectra were acquired by Microflex™ mass spectrometer (Bruker Daltonik), with a mass range of 2–20 kDa, using flexControl™ 3.0 software (Bruker Daltonik). Spectra were loaded into MALDI BioTyper™ 3.0 (Bruker Daltonik), using default settings, and compared with the manufacturer’s library. Standard Bruker interpretative criteria were applied; scores ≥ 2.0 were accepted for species assignment and scores ≥1.7 but ≤2.0 for identification at the genus level.

### 16S rRNA sequencing and phylogenetic analysis

The subspecies identification was achieved through 16 S rRNA sequencing (1–1.3 kb) and phylogenetic analysis. DNA extraction was performed according to Boom *et al*.^32^ protocol with previous enzymatic treatment with lysozyme (100 mg) and proteinase K (20 mg) (US Biological, Swampscott, MA, USA) at 37 °C for 60 min. The 16 S rRNA gene amplification was performed using Twomey *et al*.^33^ primers. The Illustra GFXTM PCR DNA and Gel Band Purification kit (GE Healthcare) was used for amplicon purification and sequencing was performed by the Human Genome Research Center (Universidade de São Paulo, Brazil). The phylogenetic analysis was performed with Mega 5.10 software using the maximum-likelihood method, and 1000 bootstrap replicates were used for branch support statistical inference. All DNA sequences from this study were deposited in GenBank under accession numbers MF113276 - MF113287.

### SE-AFLP genotyping

The obtained isolates were further genotyped by single enzyme amplified fragments length polymorphism (SE-AFLP) following McLauchlin *et al*.^34^ protocol. DNA fragments were detected by electrophoresis at 24 V for 26 h in 2% agarose gel stained with BlueGreen® (LGC Biotecnologia, São Paulo, Brazil). Fingerprint patterns were analyzed by comprehensive pairwise comparisons using Dice coefficient. A dendrogram was generated by Bionumerics 7.6 (Applied Maths, Saint-Martens-Latem, Belgium) and a 90% genetic similarity cut-off value was applied for cluster analysis^35^.

## Electronic supplementary material

Supplementary Figure S1
