# Author Correction: Septicemia due to *Streptococcus dysgalactiae* subspecies *dysgalactiae* in vampire bats (*Desmodus rotundus*)

**DOI:** 10.1038/s41598-018-29407-5

**Published:** 2018-07-19

**Authors:** Mateus de Souza Ribeiro Mioni, Fernando Favian Castro Castro, Luisa Zanolli Moreno, Camila Michelle Apolinário, Lais Dario Belaz, Marina Gea Peres, Bruna Letícia Davidé Ribeiro, Maria José da Silva Castro, Adriano Martison Ferreira, Adriana Cortez, Andrea Micke Moreno, Marcos Bryan Heinemann, Jane Megid

**Affiliations:** 1 Universidade Estadual Paulista “Júlio de Mesquita Filho”, Botucatu, São Paulo Brazil; 2 Universidad Antonio Nariño, Popayan, Cauca Colombia; 3 Universidade de São Paulo, São Paulo, Brazil; 4 Universidade de Santo Amaro, São Paulo, São Paulo Brazil

Correction to: *Scientific Reports* 10.1038/s41598-018-28061-1, published online 27 June 2018

The original version of this Article contained an error in Affiliation 4 which was incorrectly given as ‘Universidade de Santo Amaro, Botucatu, São Paulo, Brasil’

The correct affiliation is listed below:

Universidade de Santo Amaro, São Paulo, São Paulo, Brasil

This error has now been corrected in the HTML and PDF versions of the Article.

