# Supplementary material for: Septicemia due to Streptococcus dysgalactiae subspecies dysgalactiae in vampire bats (Desmodus rotundus)
Source: Sci Rep. 2018 Jun 27;8:9772. doi: 10.1038/s41598-018-28061-1 (PMC6021443; doi:10.1038/s41598-018-28061-1)
Supplement: Supplementary file 1 — Supplementary Figure S1 [file 41598_2018_28061_MOESM1_ESM.pdf]

Title: Septicemia due to *Streptococcus dysgalactiae* subspecies *dysgalactiae* in vampire bats (*Desmodus rotundus*).

Authors: Mateus de Souza Ribeiro Mioni [1], Fernando Favian Castro Castro[2], Luisa Zanolli Moreno[3], Camila Michelle Apolinário[1], Lais Dario Belaz[1], Marina Gea Peres[1], Bruna Letícia Davidé Ribeiro[1], Maria José da Silva Castro[1], Adriano Martison Ferreira[1], Adriana Cortez[4], Andrea Micke Moreno[3], Marcos Bryan Heinemann[3] and Jane Megid[1,*]

Affiliations:

[1] Universidade Estadual Paulista Júlio de Mesquita Filho, Botucatu, São Paulo, Brasil

[2] Universidad Antonio Nariño, Popayán, Cauca, Colombia

[3] Universidade de São Paulo, São Paulo, Brasil

[4] Universidade de Santo Amaro, São Paulo, São Paulo, Brasil

* **Corresponding author:** Jane Megid, Faculdade de Medicina Veterinária e Zootecnia, Universidade Estadual Paulista Julio de Mesquita Filho - UNESP, Distrito de Rubião Júnior s/n, Botucatu-São Paulo, Brasil. Cep: 18618-970; Tel.: +55 14 3880 2103; Fax: +55 14 38802042; email: jane@fmvz.unesp.br

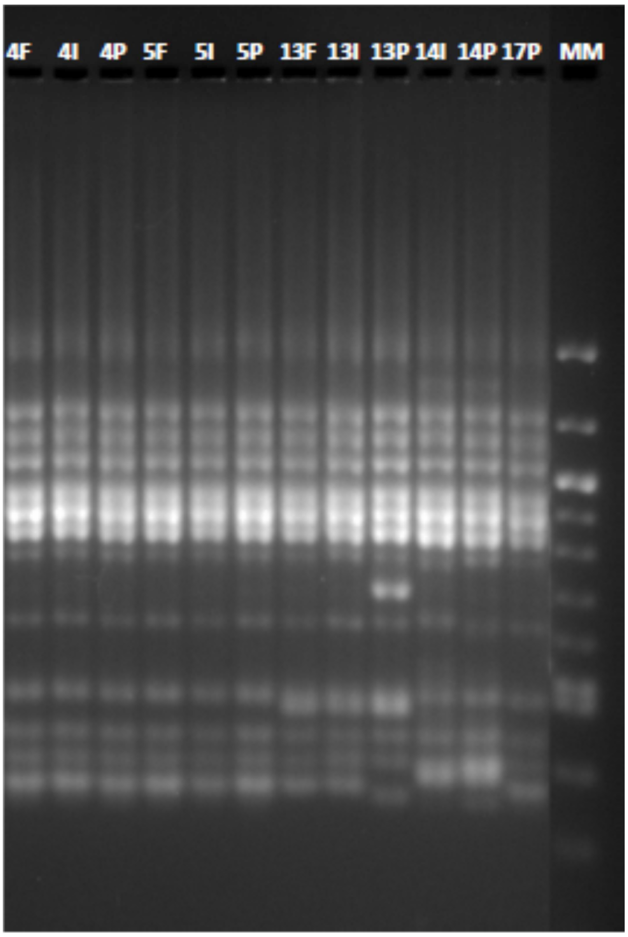

Supplementary Figure S1. SE-AFLP profiles of *Streptococcus dysgalactiae subps. dysgalactiae*. MM, 100pb molecular weight standard. The remaining lanes show AFLP profiles
